# Mass casualty incidents - time to engage

**DOI:** 10.1186/s13017-016-0064-7

**Published:** 2016-02-03

**Authors:** Offir Ben-Ishay, Michele Mitaritonno, Fausto Catena, Massimo Sartelli, Luca Ansaloni, Yoram Kluger

**Affiliations:** Surgical Oncology, Pancreatic & Hepatobiliary Surgery Service, Department of General Surgery, Division of Surgery, Rambam Health Care Campus, 8 Ha’Aliyah st., Haifa, 35254 Israel; Department of Emergency and Trauma Surgery of the University Hospital of Parma, Parma, Italy; Department of Surgery, Macerata Hospital, Macerata, Italy; Department of General and Emergency Surgery, Papa Giovanni XIII Hospital, Bergamo, Italy

## Abstract

Mass casualty incident continues to overwhelm medical systems worldwide.

Preparedness for an MCI is a crucial requisite for the injured better outcome. The World Society of Emergency Surgery initiated a survey in regard to its senior member's personal and institutional preparedness for MCI. The results here in presented indicate that WSES should engage in a formatted and structured preparedness course for medical institutions and individuals.

“*By all appearances it seems to be just another normal Saturday morning in the emergency department (ED). Patients occupy thirty out of the sixty beds; some awaits discharge, some awaits admission to the hospital. All of a sudden the squeaky voice of the red phone is tearing the air, the hard metal voice on the line is reporting of an explosion in the nearby train station, estimated number of casualties is 80. You ask for their estimated time of arrival, when you hear the first sirens of ambulances parking out of the ED; no answer was needed.*

## Background

Although intuitively the number of injured was always related to the definition of a mass casualty incident (MCI), today accepted definition refers to an event that *overwhelms* the local healthcare system, with number of casualties that vastly *exceeds* the local *resources* and capabilities in a short period of *time*. The definitions of a mass casualty incident depend on the resources of the admitting institutions. The outcome however depends on their preparedness.

Many lives could be saved in any MCI if the affected medical organizations were better prepared and an organized accessible response system already primed. Experience show that the public is the first to provide emergency assistance in such incidents. For this reason, preparedness planning increasingly emphasizes the need for building capacity (human, organizational and infrastructural) at the community level.

The common gaps in health system preparedness around the world are well documented. They are often not addressed in a comprehensive and systematic way. In particular, many countries have not yet developed Mass Casualty Management Plans, and medical systems often develop preparedness and response plans after they experienced an MCI by themselves without guidance from experienced authorities.

Along with planning, a well-designed and consistently updated training program is an essential component of a successful emergency response. Standards for training and education are required for health personal involved in mass casualty management. A great deal of information already exists to guide the setting of such standards and the design of training programs.

These information and training are delivered today by a number of professional organizations, local and national agencies. The world health organization (WHO) recognizing the existence of such deficit established a task force on MCI preparedness. In the year 2006 this task force recognized guidelines and strategies for proper building of health sector capacity in case of a mass casualty incident [1].

## Methods

During the summer of 2015, the World Society of Emergency Surgery (WSES) held its biannual conference in Jerusalem, Israel. A plenary sessions of the conference was dedicated to examine the role of the WSES in strategic planning and preparedness for an MCI. In order to understand the international needs an international survey in regard to experience with and preparedness for an MCI was initiated. The survey included 25 questions and was sent via e-mail to executive members of WSES around the world.

## Results

Forty six surgeons from 46 different institutions in 31 countries from all around the world complied with the survey. Most of the surgeons involved in the survey practice in cities of 100.000-500.000 (39.1 %) and more than 1 million (34.8 %) habitants. 56.5 % of the institutions participating in the survey were University Hospitals and 26.1 % Governmental Hospitals. Ninety one percent of the hospitals have a trauma service actively involved in trauma management and most of them (59.6 %) treat yearly between 100–500 severely injured patients (ISS >16).

Interestingly 34.4 % of the centers involved in the survey experienced more than 5 MCI, and 50 % declared managing at least 3 MCI in the past. 56.3 % were involved in MCI within the last 5 years and 34.4 % experienced one in the last 5–10 years. 50 % of the medical centers treated 10–40 injured in average during the MCI.

Not surprisingly 73.9 % of the centers had a written MCI protocols and in 67.4 % surgeons (General Surgeons or Trauma Surgeons) are directly involved in the management of such event (Table 1). Triage protocols in MCI are different when compared to routine triage [2]. Eighty three percent of the institutions reported an assigned triage officer in MCI management.

**Table 1:** Results of the survey performed by the WSES during the summer of 2015

	n-46
Written protocol for MCI (%)	73.9 (n-34)
When was it instituted (%)	
1-5 years	36.9 (n-17)
>5 years	32.6 (n-15)
What is it based on (%)	
Adaption of a protocol of another institution	19.6 (n-9)
Local Clinical experience	30.4 (n-14)
Scientific data	30.4 (n-14)
Responsible of medical management (%)	
Surgeon	32.6 (n-15)
Trauma Surgeon	28.3 (n-13)
Other	32.6 (n-15)
Assigned triage officer (%) (yes)	82.6 (n-38)
Regular MCI drills (%)	47.8 (n-22)
How often (%)	
Once a year	28.3 (n-13)
Every 2 years	13 (n-6)
>3 years	6.5 (n-3)
Who is in charge of pre hospital care (%)	
EMS	69.6 (n-32)
FD	10.9 (n-5)
Local law enforcement	6.5 (n-3)
Military	4.3 (n-2)
Familiarity with the concept of a triage hospital (%) (yes)	60.9 (n-28)
Surge capacity of your hospital (%) (yes)	60.9 (n-28)
If yes is it embedded in your MCI protocol (%) (yes)	50 (n-23)
Interhospital triage system (%) (yes)	54.3 (n-25)
Did you ever participated in an MCI course (%) (yes)	28.3 (n-13)
Would you interested in participating in one? (%) (yes)	84.8 (n-39)

Training is an integral part of MCI preparedness and management plans: it provides a setting in which operational and even medical details may be critically examined becoming an important source of feedback regarding potential problems. Furthermore, drills keep strictly in contact all professional involved in MCI developing personal and professional relationships [3, 4].

Only 47.8 % of the institutions involved in the survey perform constructed drills. Only 28.3 % of them perform trainings once a year and 13 % every 2 years. These figures pose a great concern in regard to preparedness maintenance of any size and experienced medical facility.

Although MCI management courses are available around the world only 28.3 % of the responders have participated in such a course. Eighty five percent are extremely interested in joining such an enterprise.

## Discussion

With no doubt mass casualty incidents are of an immense importance and relevance today more than ever. If it is for natural disasters, transportation accidents or manmade terror attacks; the problem remains the same, a number of casualties that overwhelms the local health care system in a very short period of time. Although the importance is well understood still some institutions around the world are not well prepared as the current survey shows. Preparedness courses are offered by various institutions such as FEMA (Federal emergency management agency), EMS (Emergency medical services) and local courses in various institutions around the world but many of these courses are not readily available for surgeons internationally.

Although the setup is different in every institution, the basics are quite the same. WSES can establish an educational platform to share this information and to create a basic course on MCI related issues such as preparedness and management. WSES through the involvement in such an enterprise can offer onsite visits to evaluate the local setup and needs as well as to conduct together with local authorities MCI trainings. The survey shows that only 28.3 % participated in constructed course while 84.3 % are very interested in participating in one.

## Conclusion

In conclusion, the vast unfortunate experience of many countries around the world did not translate into a massive preparedness of hospitals towards a shattering event such as an MCI. Although half of the centers involved in the survey reported managing up to 3 MCI's in the last 5 years only one third of the responders participated in an MCI management course. We believe that international need and the thirst for knowledge of surgeons around the world creates an opportunity for WSES to engage in an initiative in regard to MCI management course and on site preparedness drills.

WSES has the capabilities and personal to create a uniform MCI management course and to take a role in the international preparedness for such an event.
